# Enhanced Bone Regeneration Using Demineralized Dentin Matrix: A Comparative Study in Alveolar Bone Repair

**DOI:** 10.1016/j.identj.2025.03.026

**Published:** 2025-05-03

**Authors:** Nessma Sultan, Bassant Mowafey, Fatma Ata, Mona H. El-Zekrid, Soher Nagi Jayash

**Affiliations:** a Faculty of Dentistry, Mansoura University, Mansoura, Egypt; b Faculty of Dentistry, Mansoura National University, Gamasa, Egypt; c Faculty of Dentistry, Mansoura University, Egypt; d Roslin Institute, University of Edinburgh, Edinburgh, UK

**Keywords:** Demineralised dentin matrix, Alveolar ridge augmentation, Bone regeneration, Cone-beam computed tomography, Immunohistochemistry

## Abstract

**Objectives:**

Alveolar bone resorption following tooth extraction presents significant challenges for implant-supported rehabilitations. Demineralised dentin matrix (DDM) has emerged as a promising scaffold for bone tissue regeneration. This study evaluates the bone-regenerating potential of varying degrees of dentin demineralisation.

**Materials and methods:**

Thirty-two male white New Zealand rabbits underwent extraction of the left mandibular anterior tooth and were assigned to 4 groups: undemineralised dentin matrix (UDDM), partially demineralised dentin matrix (PDDM), completely demineralised dentin matrix (CDDM), and a control group with no treatment. At 4 and 8 weeks post extraction, cone-beam computed tomography (CBCT) was used to assess alveolar bone height and width. Histological analyses using H&E and Masson trichrome stains evaluated new bone formation, and immunohistochemistry detected osteopontin expression.

**Results:**

CBCT imaging revealed progressive increases in alveolar bone height and width across all groups over time. Histological analysis showed new bone formation in all groups, with the PDDM group demonstrating closer integration of newly formed bone trabeculae compared with the others. IHC results showed higher osteopontin expression in the PDDM group, highlighting its superior bone-inductive potential.

**Conclusion:**

Among the tested materials, PDDM exhibited the most effective bone induction and tissue regeneration capabilities, outperforming CDDM and UDDM in promoting alveolar bone repair. These findings position PDDM as a valuable scaffold for enhancing bone tissue regeneration in clinical applications.

**Clinical relevance:**

The use of PDDM in tooth extraction sockets significantly promotes efficient and reliable bone regeneration, making it a valuable option for clinical applications in implant dentistry.

## Introduction

When planning for future rehabilitation, it is important to consider the short- and medium-term morphological and physiological alterations that tooth extraction can occasionally cause at the alveolar bone level.^1^^,^^2^ The primary goal of alveolar preservation approaches following tooth extraction is to minimise the socket's vertical and horizontal structural alterations, with the ultimate goal of having more bone volume after the healing process is completed. This will enable a prosthetic rehabilitation that is as aesthetically pleasing and functional as possible following the insertion of an osseointegrated implant. In spite of everything, a certain amount of reabsorption cannot be avoided, even with the methods for alveolar preservation; this will happen regardless of the approach employed.^3^^,^^4^

Bone-grafting techniques, particularly alveolar ridge augmentation, have advanced the feasibility of placing implants in previously unsuitable sites. Among grafting materials, autogenous bone grafts are considered the gold standard due to their superior osteogenic potential. However, their use is often limited by donor site morbidity and the associated risk of complications. To overcome these challenges, nonautogenous graft materials have gained popularity for their ability to support osteoconduction and osteoinduction. Such materials provide a scaffold for osteogenic cells while converting mesenchymal cells into bone-forming cells, enhancing bone regeneration.^5^, ^6^, ^7^, ^8^

Dentin, comprising more than 80% of tooth structure, is a promising material for bone regeneration. Its mineral-rich composition and noncollagenous proteins, such as phosphoproteins, osteocalcin, and osteopontin (OPN), play a vital role in bone architecture development.^4^ Notably, dentin shares key characteristics with autogenous grafts: it is osteocompatible and osteoconductive. The inorganic component of dentin acts as a scaffold for new bone formation while its organic portion delivers essential growth factors. Additionally, dentin’s lack of osteocytes and blood vessels minimises the risk of infection and immunogenicity, making it a safe and effective biological material for tissue regeneration.^9^, ^10^, ^11^

Demineralised dentin matrix (DDM) is particularly noteworthy for its potential for bone regeneration. DDM consists of acid-insoluble collagen with an ultra-fine structure formed by dentinal tubules. Its cell-free matrix and absorbable extracellular components offer structural support crucial for implant stability. Moreover, DDM retains bioactive bone morphogenic proteins (BMPs), such as BMP-II, BMP-IV, and BMP-VII, even after demineralisation, which further enhances its osteoinductive properties.^7^^,^^8^

The growth factors and proteins necessary for tissue regeneration and repair must be released through the demineralisation process. However, by eliminating part of the highly crystalline inorganic materials from the dental hard tissues, the supporting collagen matrix collapses and degrades, causing the dental hard tissues to lose their structural integrity. Certain acids and chelating chemicals, such as 17% ethylenediaminetetraacetic acid (EDTA), sodium hypochlorite, and citric acid, were used for the demineralisation process. Citric acid produced the most damaging effect, whereas EDTA had different effects on collagen.^12^ Previous data showed that EDTA is an effective demineralising agent for the production of DDM, enabling minimal or even no loss of structural integrity. By comparison, strong acids like HCl have been found to produce a more degraded and detached tubular structure even at lower concentrations.^13^

The degree of demineralisation is critical for optimal dentin regeneration. The partially demineralised dentin matrix (PDDM) results in the elimination of the major part of the mineral phase and immunogenic components while retaining a very low fraction of minerals (5-10 wt%), whereas the completely demineralised dentin matrix (CDDM) results in complete elimination of minerals while retaining the major organic component of the dentin matrix.^14^

CDDM has demonstrated biocompatibility and antimicrobial effects^14^; however, little is known about the comparative efficacy of CDDM, PDDM, and undemineralised dentin matrix (UDDM) in promoting alveolar bone regeneration. This study aims to assess the osteoinductive potential of DDM with varying degrees of demineralisation. Specifically, we aim to evaluate the ability of PDDM, CDDM, and UDDM to enhance alveolar bone development following tooth extraction. Importantly, this is the first study to investigate the impact of demineralisation levels on DDM activity, providing valuable insights into its clinical application for alveolar bone augmentation.

## Materials and methods

### Preparation of demineralised dentin matrix

Ten permanent teeth (*n* = 10) free of carious lesions were taken from the Mansoura University Faculty of Dentistry's outpatient clinic. These teeth were extracted for orthodontic purposes and subsequently used in the study. The Faculty of Dentistry Ethical Committee adopted infection control guidelines for the collection and storage of teeth. Any remaining soft tissues around the teeth were excised, and enamel and cementum were removed by a high-speed contra angle handpiece. The collected teeth were crushed into particles by a miller and sieved using a sieve system to obtain particle sizes of 300 to 500 μm.^15^^,^^16^ To maximise the preservation of the bioactive organic component, the milled dentin particles were treated with 17% EDTA at pH 7.0 and applied at different time periods.

Three groups (10 g each) were randomly selected from the dried dentin particles: group I (UDDM, or control); group II (PDDM; demineralised for 20 minutes); and group III (CDDM; demineralised for 3 days).^14^ Following incubation, the EDTA was discarded, the specimens were rinsed twice in phosphate-buffered saline (PBS), and the particles were dried and sterilised with ultraviolet light for 15 minutes before being stored at 4 °C.

### Characterisation of the demineralised dentin particles

All characterisation was conducted before in our previously published work.^14^ Chemical composition of the UDDM, PDDM, and CDDM particles was analysed by scanning electron microscopy and energy-dispersive x-ray analysis. Enzyme-linked immunosorbent assay was used for quantification of collagen-I, bone morphogenetic protein-2, and vascular endothelial growth factor in UDDM, PDDM, and CDDM particles.

### Surgical procedure

Thirty-two male, white New Zealand rabbits weighing 2 to 2.5 kg each were used. Operation and maintenance were carried out at the experimental research centre of the Faculty of Medicine, Mansoura University. The animals were housed in separate cages under optimal experimental conditions based on the protocols approved by the animal ethics committee under the reference number A25060722. The animals were fed a standard pellet diet and tap water throughout the study period. The animals were subjected to cycles of 12 hours of light and 12 hours of dark at a temperature of 22 °C.

At the time of surgery, general anaesthesia was induced via intramuscular injection of Zoletil 50 (0.5 mL/kg) (Virbac) and Xylazine (0.25 mg/kg) (Rompun). The surgery was performed in the anterior area of the lower jaw. The lower left central incisor was luxated with a small elevator and extracted using paediatric lower anterior forceps. After the socket’s integrity was examined, the site was divided into the tested groups (which had the socket filled with the tested biomaterials using a Malt surgical curette) and the control group (which underwent tension-free suture healing).

### Study design

#### Sample size calculation

Eight rabbits were assigned to each group. In each group, 4 rabbits were sacrificed at 4 weeks and the remaining 4 at 8 weeks post treatment. The group size was determined through power analysis based on our previously published histological data, aiming for a power of 95% and a significance level of 5%. According to prior knowledge, 4 defects per group per time point were deemed sufficient for statistical analysis.^17^

The experimental groups were categorised as follows:

Group I (Control): The socket was left empty.
Group II (UDDM): The socket was filled with the undemineralised dentin matrix.
Group III (PDDM): The socket was filled with the partially demineralised dentin matrix.
Group IV (CDDM): The socket was filled with the completely demineralised dentin matrix.

**Fig. 1:**
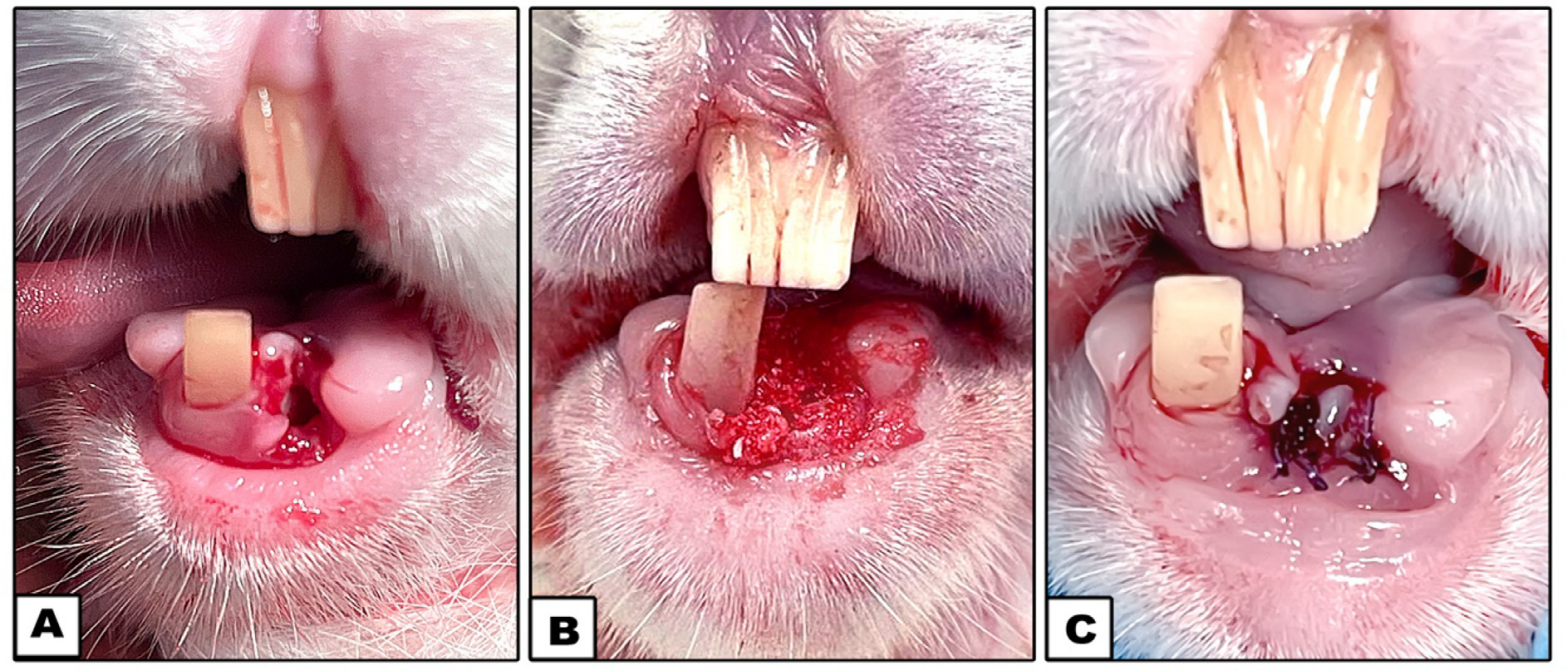
Photographs showing the lower left incisor socket (A), filling the socket with DDM (B), and the overlying soft tissues sutured in layers (C).

For the treated groups (Groups II-IV), 20 mg of DDM was used to fill each socket (Figure 1).

#### Postoperative care

The covering soft tissues were sewn in layers using absorbable 3-0 Vicryl sutures (Ethicon). Using sterile gauze, the incision site was cleansed with an antiseptic solution containing povidone-iodine. Analgesics (Voltaren 75 mg/3 mL) and antibiotics (cefotaxime 1 g) were given to each experimental animal every 24 hours for 3 days after surgery.^18^

#### Euthanasia

At 4 and 8 weeks following surgery, animals were sacrificed by an overdose injection of sodium thiopental (60 mg/kg) and then their mandibles were harvested for radiographic and histologic assessment.

### Scanning electron microscopy and energy-dispersive x-ray analysis

To assess the surface morphology and chemical structure of the dentin particles, a scanning electron microscope (SEM) (Jeol JSM 6510) was used attached to a secondary electron detector for energy-dispersive x-ray analysis (Oxford X-Max 20).

Following gold sputtering, the dentin particles were mounted onto SEM stubs and properly assessed by using an accelerating voltage of 15 kV. The size of dentin particles and the impact of different levels of demineralisation on dentin structure were shown in a previously published work.^14^

### Radiographic analysis

**Fig. 2:**
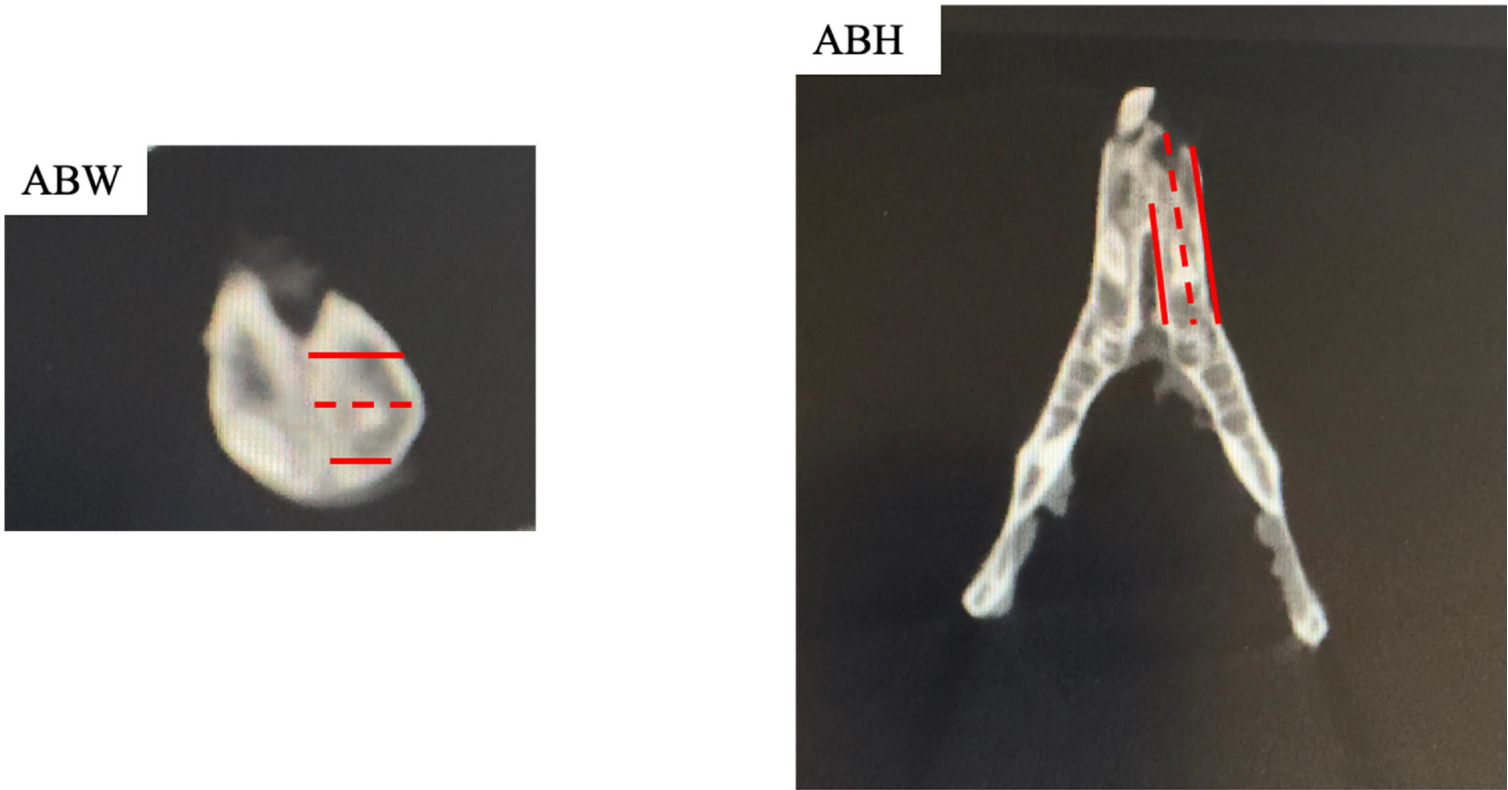
CBCT micrograph showing schematic measurements of ABW and ABH.

After the animals were sacrificed at 4 and 8 weeks following surgery, CBCT images was acquired. Qualified radiologists fastened each mandible to the CBCT chair and then adjusted it to the proper height to finish the CBCT scan. The same radiologist acquired all sectional pictures by using a CBCT scanner (i-CAT 17–19) with the following parameters: axial slice thickness of 0.2 mm, exposure at 5.0 mA and 120 kV for 9.6 seconds. The same radiology specialist (blinded to the groups) processed and analysed the data using image analysis software. Alveolar bone height (ABH) and alveolar bone width (ABW) changes were noted. For every CBCT, 3 portions were chosen for measurement of the ABH and ABW, respectively. ABW was calculated using Chen et al.’s approach.^19^ Cross-sectional slices in the apical, middle, and coronal thirds of the socket were measured. An earlier approach by Liu et al. was used to measure ABH.^20^ Three sagittal planes were measured: the lingual plane, the buccal plane of the extraction socket, and the middle plane of the initial 2 planes (Figure 2).^21^ The measured CBCT values were used to express changes in ABW and ABH.

### Radiographic measurements

Two assessments of the measurements were conducted. An oral radiologist with 5 years of experience viewed all the images on the same 32-inch big screen with high resolution in low light. A second radiologist, with 15 years of experience, provided advice on how to interpret the images with unexpected variances. Measurements were checked twice with 2-week intervals between the 2 sessions. An Excel 2013 (Microsoft) table was used to record the data, which were then tracked and statistically examined.

### Histological examination

Following a radiological assessment, the specimens were ready for histological analysis by following standard protocols. In brief, the samples were decalcified in 0.5 M EDTA (pH 7.8) and fixed with 4% paraformaldehyde in 0.1 M PBS (pH 7.4). Following decalcification, every specimen was split into 2 blocks precisely in the middle of the initial surgical defect and embedded in paraffin. Transverse serial slices each 3 μm thick were then created. The sections were stained with haematoxylin and eosin (H&E) and Masson trichrome (MT) (Cat#HT15, Sigma Aldrich). Histologic assessment was conducted using an Olympus BX-51 optical microscope.

### Immunohistochemistry

The osteopontin antibody (polyclonal antirabbit osteopontin (OPN); Cat# ES3083 at a 1:150 dilution, ELK Biotenchnology) was used for the immunohistochemical analysis, which was conducted via a traditional streptavidin-biotin immunoperoxidase approach. Following paraffin embedding, 3-μm-thick histologic sections were cut, deparaffinised, and hydrated. The formalin pigments were removed by soaking the sample for 10 minutes in a mixture of 5% ammonium hydroxide and 95% ethyl alcohol. The sample was washed twice, first under tap water and then under distilled water. To remove antigens, the tissue slices were treated for 60 minutes at 37 °C with 1% pepsin solution (pH 1.8). Next, 3% hydrogen peroxide was used to clean and block the samples.

After that, the specimens were cleaned under tap water and twice more with distilled water to remove the solution. To avoid significantly altering the pH, the tissue sections were promptly incubated at pH 7.4 in BSA. The primary antibody, polyclonal OPN, was diluted 1:150 in PBS solution comprising 0.1% NaN3 and 1% BSA. The slides were then left to incubate at 4 °C for 12 hours in a moisture chamber. Instead of incubating with the primary antibody, the same sections were used as a negative control.

The samples were incubated in a dark chamber for 3 minutes using 300 mg of diaminobenzidine (also known as 3,3-diaminobenzidine; Sigma Chemical) dissolved in 100 mL of pH 7.4 PBS solution. After haematoxylin counterstaining, the slides were washed for 3 minutes. After being cleaned in xylene and dried with an increasing ethanol series, the slides were mounted on Permount (Fischer Scientific) for light microscopic analysis.

### Histomorphometric analysis

At 4 and 8 weeks following tooth extraction, 4 tissue slides from each group were made. Using a 100× magnification, 4 randomly selected locations from the defect (central area) were used to create microscopic images of the extraction sites. Next, using 16 images per group each week, the ratio of recently formed bone to the whole defect area in the slide images was measured. The Intel Core I7 computer and VideoTest Morphology software (Version 5.0), which has a built-in procedure for area and percentage area measurements, were used to conduct this investigation. The images used for analysis were stained with MT and OPN. Regenerated bone, collagen fibres, or osteoid could be detected by the development of a bluish discoloration with MT staining. Positive reactions could be detected by the development of brown discoloration with OPN immune staining.

### Statistical analysis

Statistical analysis was conducted by using SSPS version 17.0 via 1-way ANOVA followed by Tukey post hoc analysis. The *t* test was employed for pairwise comparisons of the quantitative data that the Kolmogorov-Smirnov test determined to be normally distributed.

Intraclass correlation coefficients (ICCs) were used to evaluate the reliability of repeated radiographic measurements. All assessments made by the same radiologist of the randomly chosen CBCT images at a 2-week interval between the 2 assessment sessions showed excellent reliability with ICCs of at least 0.92. Details of the statistical tests used for each experiment are provided in the respective figure legends. The significance level was set at *P* < .05.

## Results

### Clinical findings

The experimental animals survived the procedure and exhibited no signs of wound dehiscence, fracture, or infection at the surgical sites throughout the study. By the eighth week, there were no signs of infection, haematoma, or necrosis at the defect sites. The bone defect locations did not exhibit any clinical signs of inflammation, scarring, or adverse tissue response to the grafting materials. All the rabbits were able to feed following the treatment and return to their presurgical life after recovering from anaesthesia. They also showed no signs of discomfort or infection, such as an exudate or a red, hot incision, during the healing process.

A total of 32 defects were evaluated through histological and histomorphometric assessments, providing comprehensive insights into the bone regeneration process.

### Radiographic findings

The results of the radiographic analysis describing the alteration of ABH and ABW at 4 weeks and 8 weeks in different groups are shown in Table 1, Table 2.

**Table 1:** Alveolar bone height and width 4 weeks post surgery (*n* = 4).

Group	ABH (SD) (mm)	ABW (SD) (mm)
Control	0.78 (0.012)	0.4 (0.01)
UDDM	0.85 (0.054)	0.52 (0.026)^a^
PDDM	1.16 (0.13)^a^^,^^b^	0.62 (0.03)^a^^,^^b^
CDDM	1.01 (0.1)^a^^,^^b^^,^^c^	0.59 (0.023)^a^
*P*	<.05	<.05

ICCs = 0.92.

a Significant difference compared to the controls (*P* < .05).

b Significant difference compared to the UDDM group (*P* < .05).

c Significant difference compared to the PDDM group (*P =* .03).

**Table 2:** Alveolar bone height and width 8 weeks post surgery (*n* = 4).

Group	ABH (SD) (mm)	ABW (SD) (mm)
Control	0.78 (0.021)	0.4 (0.014)
UDDM	1.31 (0.47)^a^	0.69 (0.025)^a^
PDDM	1.5 (0.127)^a^^,^^b^	0.9 (0.073)^a^^,^^b^
CDDM	1.457 (0.13)^a^^,^^b^	0.845 (0.073)^a^^,^^b^
*P*	<.05	<.05

ICCs =0.92.

a Significant difference compared to the controls (*P* < .05).

b Significant difference compared to the UDDM group (*P* < .05).

In the postoperative period, the ABW and ABH in each group demonstrated various grades. Four weeks after surgery, ABW revealed a significant reduction in the control group compared with the remaining 3 groups (*P* < .05). In contrast, ABH scored significantly lower in the control group compared with PDDM and CDDM groups, but there was no significant difference in ABH changes between the control group and the UDDM group. There was a significant difference between both the CDDM and PDDM groups when compared to the UDDM group (*P* < .05). Of note, there was a significant difference in ABH changes between the CDDM group and the PDDM group (*P* = .03).

Postoperatively after 8 weeks, both ABW and ABH showed statistically significant differences between the control group and the other 3 groups (*P* < .05). Both the PDDM and CDDM groups showed a significant difference compared to the UDDM group (*P* < .05). PDDM showed higher values (ABH 1.16 [SD 0.13] after 4 weeks and 1.5 [SD 0.127] after 8 weeks; ABW 0.62 [SD 0.03] after 4 weeks and 0.9 [SD 0.073] after 8 weeks) in comparison to CDDM (ABH 1.01 [SD 0.1] after 4 weeks and 1.457 [SD 0.13] after 8 weeks; ABW 0.59 [SD 0.023] after 4 weeks and 0.845 [SD 0.073] after 8 weeks) but without being statistically significant.

### H & E analysis

#### After 4 weeks

**Fig. 3:**
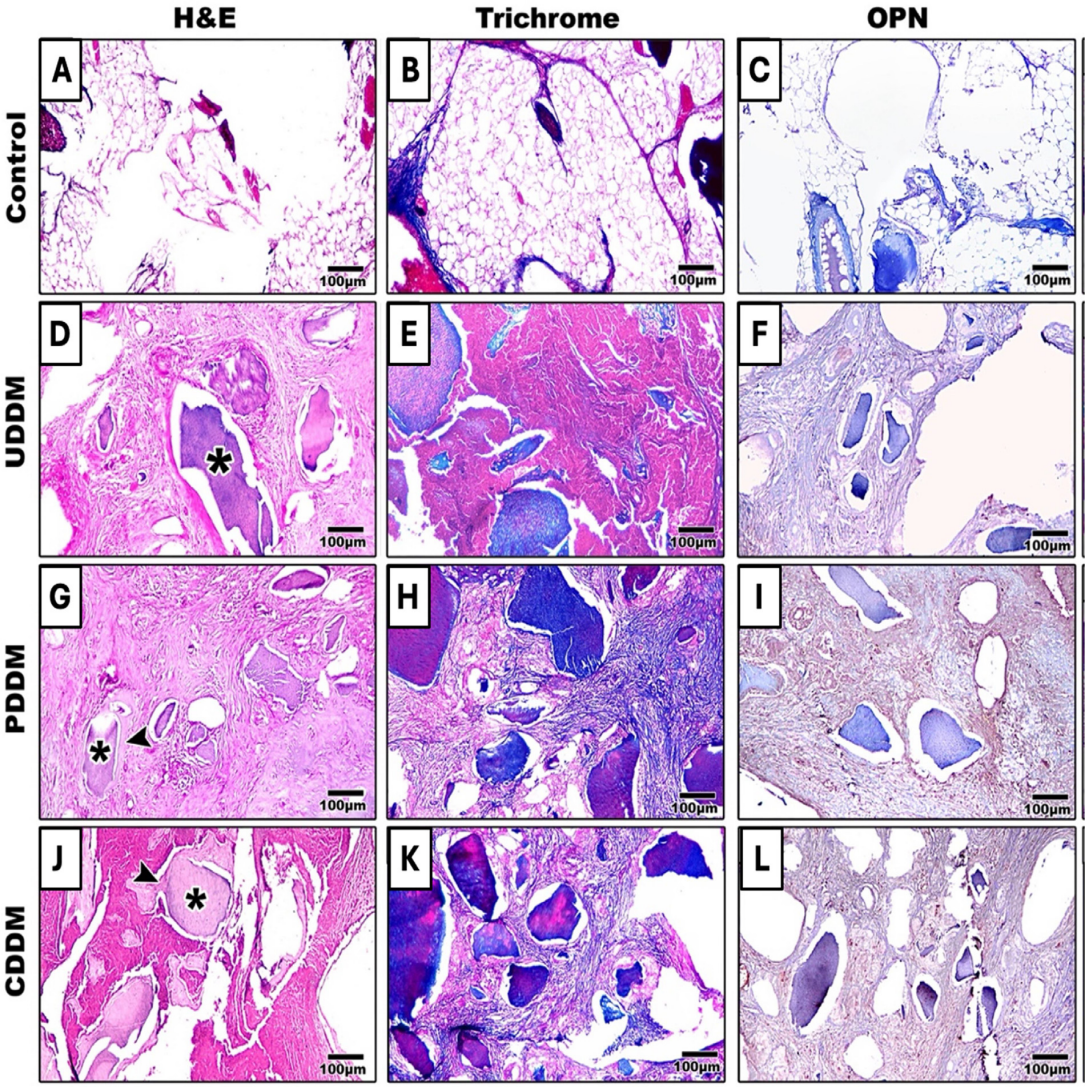
Representative histologic images of H&E (A, D, G, J), Masson’s trichrome (B, E, H, K), and osteopontin immune staining (C, F, I, L) at 4 weeks post treatment. In the control group, the defects are seen to be filled mainly with fatty tissue (A-C). The UDDM group shows dentin particles (*) with fibrous tissue surrounding (D-F) with no newly formed bone. The PDDM group shows smaller (resorbed) dentin particles (*) with osteoid bone surrounding the particles (arrowhead) and dense fibrous connective tissue filling the defect area (G-I). In the CDDM group, dentin particles started to resorb (*) surrounded by osteoid tissue (arrowhead) with irregularly arranged fibrous tissue around them (J-L). Scale bar: 100 μm.

In the control group, adipose tissue filled the extraction socket with no new bone observed. In the PDDM group, there were abundant osteoblasts that were arranged in rows adjacent to the bony matrix, and mature fibrous tissue could be detected. PDDM were surrounded by newly formed bone (osteoid), and direct contacts were noticed between the DDM and the new bone in both PDDM and CDDM groups, demonstrating osteoconductive properties of the DDM. CDDM displayed good tissue integrity, but the arrangement of fibrous connective tissue was irregular, with blood vessels growing into the extraction sockets. As for UDDM, the socket was occupied by fibrous connective tissue with no new bone formation observed (Figure 3 A, D, G, J).

#### After 8 weeks

**Fig. 4:**
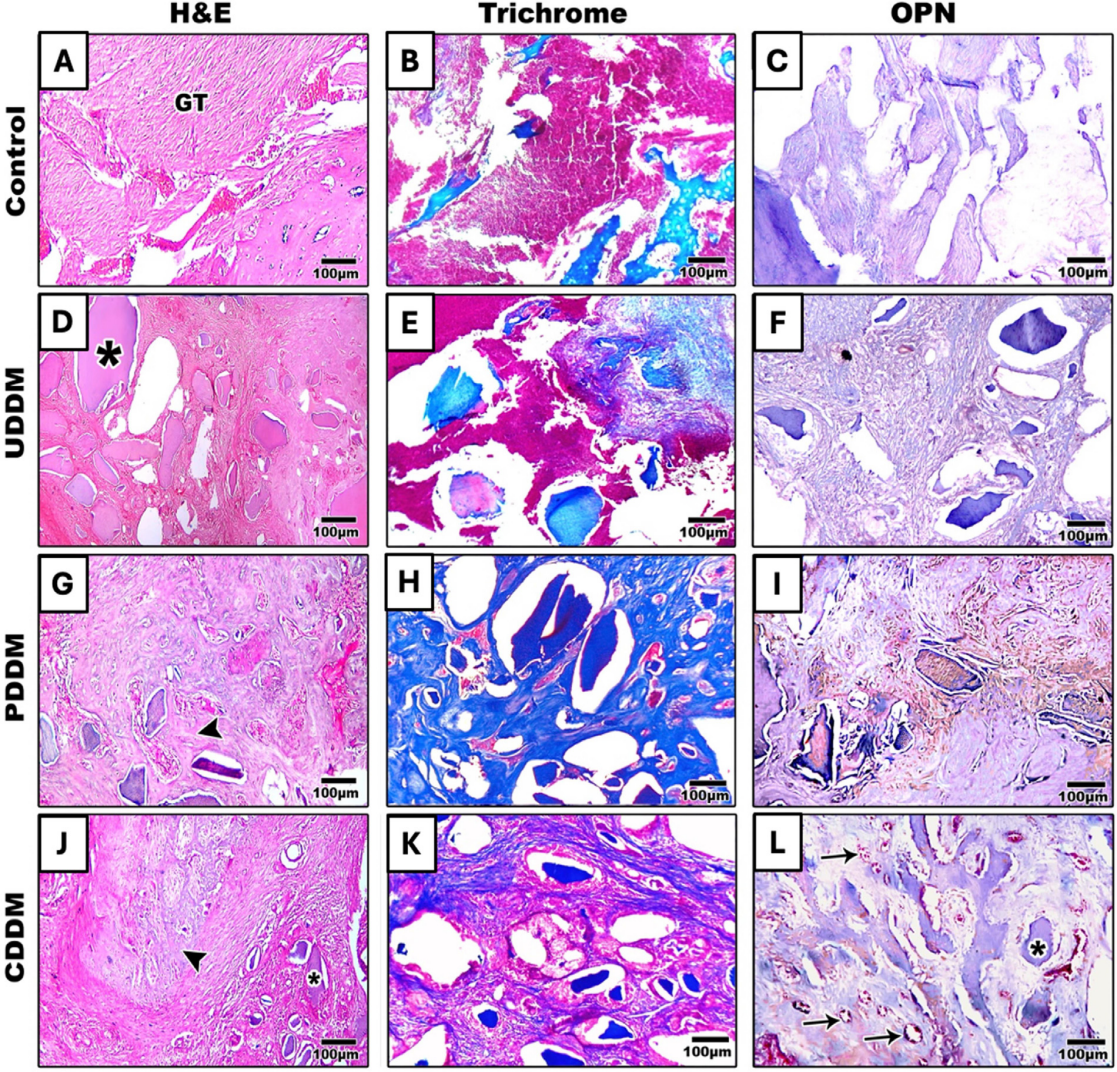
Representative histologic images of H&E (A, D, G, J), Masson’s trichrome (B, E, H, K), and osteopontin immune staining (C, F, I, L) at 8 weeks post treatment. In the control group, the defect area is filled mainly with granulation tissue (A-C). The UDDM group shows dentin particles (*) with well-aligned surrounding fibrous tissue (D-F) with no newly formed bone. The PDDM group shows smaller dentin particles (*) with newly formed bone trabeculae surrounding them (arrowhead) (G-I). In the CDDM group, the markedly resorbed dentin particles (*) are surrounded by relatively thin newly formed bone trabeculae with dense fibrous tissue with abundant vasculature (arrow) (J-L). Scale bar: 100 μm.

Each experimental group had grown more new bone by the eighth week compared to the controls. The extraction site in the control group was filled with well-organised connective tissue. In the PDDM group, a small volume of fibrous connective tissue and dentin graft particles was still visible, but new bone had formed in the extraction alveolus. The new bone trabeculae were thick and calcified, strictly united, and arranged similarly to the normal condition. Of note, in the PDDM group, the defect was fully filled and completely closed with newly formed bone. The new bone had grown and continued to spread towards the centre of the sockets. Osteoblast proliferation was observed, the bone trabeculae had increased in number, the new bones were attached to each other, and the fibrous connective tissue and inflammatory cells had diminished. Although there was some new bone development in CDDM, it was noticeably less than in the PDDM group. The extensive vasculature observed in the CDDM group may have contributed to the fact that the dentin graft underwent faster resorption in this group than in the other experimental groups. The new bone in the extraction sockets in the UDDM group was thin and confined to the socket's edge (Figure 4 A, D, G, J).

### Masson trichrome staining results

#### After 4 weeks

**Fig. 5:**
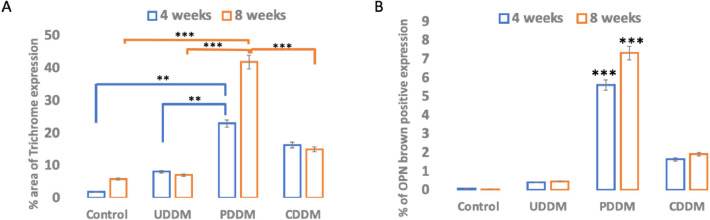
Histomorphometric analysis of MT (A) and OPN (B) shows significantly higher expression in the PDDM group compared to other groups at both 4 and 8 weeks (*P* < .01 and *P < .001, respectively, indicate statistically significant differences).

The 3 treated groups had a more prominent blue colouration, which is indicative of collagen deposition, when compared to the controls (Figure 3 B, E, H, K). This difference clearly suggested that 4 weeks after surgery, bone matrix formation was noticeably less active in the control group (1.905 [SD 0.005]). On the other hand, in the UDDM, PDDM, and CDDM groups, collagen deposits were obviously present at 4 weeks, representing an early and active stage of bone matrix synthesis, with percentages of 8.14 (SD 0.002), 22.92 (SD 0.01), and 16.32 (SD 0.0007), respectively. Of note, the collagen deposition significantly increased in the PDDM group compared to the UDDM and control groups, but there was no significant difference between PDDM and CDDM after 28 days postsurgically (*P* > .05) (Figure 5A).

#### After 8 weeks

A significant difference in collagen expression was observed between the UDDM and CDDM groups, further highlighting the varied effectiveness of the different treatments (Figure 4 B, E, H, K**)**. Masson trichrome staining demonstrated increased collagen deposition in all treated groups compared to the control group, which exhibited minimal collagen presence (5.9 [SD 0.0004]). Among the treated groups, the UDDM group showed a modest increase in collagen deposition (7.13 [SD 0.0008]). The PDDM group, however, exhibited the highest level of collagen deposition (41.8 [SD 0.0004]), significantly exceeding both the UDDM and CDDM groups at 4 and 8 weeks (*P* < .01 and *P* < .001, respectively). The CDDM group showed intermediate collagen deposition (14.98 [SD 0.0004]), which was notably greater than in the UDDM group (Figure 5A).

### Immunohistochemistry analysis

The volume of new bone formation (n-bone%; the brown positive expression of OPN) was measured in a quantitative manner. The mean n-bone% in the control group was 0.09 (SD 0.0004) after 4 weeks and 0.045 (SD 0.0004) after 8 weeks; in the UDDM group 0.41(SD0.004) after 4 weeks and 0.47 (SD 0.0005) after 8 weeks; in the PDDM group 5.61 (SD 0.0004) after 4 weeks and 7.33 (SD 0.0005) after 8 weeks; in the CDDM group 1.64 (SD 0.0004) after 4 weeks and 1.91 (SD 0.008) after 8 weeks. A significant difference in n-bone% was noticed between the PDDM group and the other groups after 4 and 8 weeks postsurgically (*P* < .001) (Figure 5B).

## Discussion

Mineralised or demineralised dentin grafts can be made easily from extracted teeth. The collagen matrix is revealed by demineralisation, and growth factors are released, enhancing the body's ability to regenerate. When compared to freeze-dried bone, which has been used widely in alveolar bone augmentation several years ago but carries the possibility of viral disease transmission, the allogeneic application of human DDM differs in a number of ways. Numerous tissue banks employ demineralisation, which has been shown to be an effective technique for viral inactivation, to verify the removal of viruses.^22^^,^^23^

DDM is a useful scaffold material in the field of tissue engineering. It is appropriate for promoting growth and differentiation of different cells because of its biological characteristics and natural composition. Growth factors that encourage the development of new mineralised tissues, such as bone and dentin, are thought to be the cause of DDM’s therapeutic effectiveness in regenerative dentistry.^13^ The increased collagen content (90%) of dentin as a graft material is advantageous because it has been shown to improve cell attachment and adhesion.^24^

DDM can stimulate new bone formation better than commercial bovine mineralised bone grafts such as Bio-Oss. Notably, DDM has been shown to encourage a greater rate of bone remodelling and has been found to disintegrate more quickly than Bio-Oss, according to a recent comprehensive evaluation of clinical trials.^25^

The quantity of mineral percentage that remains in the DDM is influenced by the varied demineralising agent types and preparation times used in different investigations. The inorganic/organic ratios of the DDM components differ from those of the dentin matrix's initial composition. In our previously published article,^14^ standardisation of demineralisation protocol for dentin particles with further investigations of organic and inorganic content following the different demineralisation periods was conducted. Adjusting the demineralisation process's degree is essential because otherwise it may result in inadequate osteoconduction and elimination of growth factors.

This study used particles from 300 μm to 500 μm in size. Previous research has examined the relation between particle size and the DDM's effectiveness.^26^ Increased osteoinductive activity and improved cellular responses have been linked to smaller particle sizes. They are thought to be more effective because they offer more surface area for cellular interactions, which promotes cell migration, adhesion, and proliferation.

With sufficient resolution and extremely accurate 3-dimensional radiographic images, CBCT scanning can offer crucial assessment information at a comparatively low radiation dosage and cost, allowing physicians to analyse bone alteration and changes.^27^ In the radiographic assessment conducted in this study, there were significant differences in ABH and ABW between the 4 experimental groups at both 4 and 8 weeks post surgery. The PDDM group demonstrated the most favourable results, followed by the CDDM and UDDM groups. The degree of demineralisation is key to achieving optimal bone regeneration, with PDDM showing the most promise in this regard. Partial demineralisation retains a small percentage of minerals while removing most of the mineral phase and immunogenic components. This results in an osteoconductive and osteoinductive scaffold that contains various growth factors that support bone regeneration.^12^

In histological and immunohistochemical assessments, PDDM likely promotes more osteogenic activity than CDDM, which probably could be explained by multiple noncollagenous proteins that are leached from the dentin matrix during partial demineralisation. These proteins, including osteogenic growth factors, may play a critical role in stimulating osteoblast differentiation and enhancing bone formation. This could explain why bone growth was more pronounced in the PDDM group compared to that in the CDDM group.^28^ Recent studies have concluded that PDDM is an effective bony replacement for regenerating calvarial bone defects in rabbits.^17^ Our study, however, focused on the osteogenic differences between PDDM, CDDM, and UDDM for alveolar bone augmentation. We found that full demineralisation of dentin particles tends to reduce new bone formation.^29^ A study by Turonis et al. showed that demineralised human bone grafts significantly improved osseous wound healing in rats' calvarium compared to nondemineralised bone grafts.^30^ This suggests that the mineralised nature of bone or dentin grafts may hinder the remodelling process by limiting the exposure of growth factors. Conversely, a study by Elfana et al. showed no significant difference between mineralised and demineralised dentin grafts in terms of efficacy for extraction socket healing.^31^

Our results showed that UDDM exhibited significantly lower bone regenerative activity compared to PDDM and CDDM, even though mineralised bone allografts have known osteoconductive properties and are used clinically.^32^ This may be owing to the differences in the fine dentin structure compared to bone, despite its similar mineral composition.^33^ However, demineralisation exposes dense collagen fibres that can enhance cell adhesion. Additionally, noncollagenous proteins, including osteogenic growth factors such as BMPs, are present in the dentin matrix and released during demineralisation, further supporting the osteogenic potential of dentin-based scaffolds.^34^, ^35^, ^36^ The widening of dentinal tubules during demineralisation may also act as a conduit for the release of these factors, facilitating osteoblast formation and differentiation.^37^

One of the interesting findings in this study was that PDDM exhibited more pronounced bone regenerative activity in the early stages of bone regeneration than did CDDM. Histological analysis showed that CDDM particles were smaller and occupied more of the space with connective tissue, whereas PDDM particles displayed better bone regeneration. Quantitative analysis of new bone formation at both 4 and 8 weeks corroborated these findings, suggesting that the resorption of CDDM occurred before significant bone formation. Osteoclastic activity was observed on both the PDDM and CDDM surfaces, indicating that the resorption of collagen fibrils and mineralised matrix is crucial for initiating osteoblastic bone development. Considering that multiple growth factors are released from the dentin matrix during the demineralisation process, it is likely that PDDM contains more growth factors that promote osteogenesis compared to CDDM. This may explain the more substantial bone growth observed in the PDDM group.

Research has highlighted the impact of particle size on bone regeneration, with smaller particles potentially offering more surface area for cellular interaction.^38^, ^39^, ^40^, ^41^, ^42^ However, there is no consensus on the optimal degree of degradation for graft materials to maximise bone regeneration. In this study, CDDM graft particles were resorbed before bone formation began, whereas PDDM particles supported osteoblast attachment and osteogenesis before complete resorption. These findings suggest that the optimal rate of matrix resorption is an important factor in achieving better bone regeneration. The balance between resorption and bone formation is crucial for optimal bone regeneration, and PDDM appears to offer the most favourable conditions for this process. Attaching osteoblasts and osteoprogenitor cells to PDDM particles may help to delay resorption and initiate bone formation, ultimately enhancing the regenerative process. Therefore, PDDM demonstrates the potential to offer superior outcomes for alveolar bone augmentation compared to CDDM and UDDM, providing an effective scaffold for bone regeneration.

## Conclusion

This study demonstrates that PDDM provides superior osteogenic potential for alveolar bone augmentation compared to both CDDM and UDDM. PDDM exhibited enhanced collagen deposition, osteoblast activity, and bone regeneration, particularly in the early stages of healing. The degree of demineralisation plays a critical role in optimising the regenerative capacity of dentin-based scaffolds, with partial demineralisation retaining sufficient growth factors and minerals to promote bone formation effectively. These findings suggest that PDDM could serve as a promising biomaterial for enhancing alveolar bone regeneration and improving the outcomes of implant-supported rehabilitations. Further studies are warranted to fully understand the long-term effects and potential clinical applications of PDDM in dental and maxillofacial surgery.

## Conflict of interest

None disclosed.
